# Single and multiple pathogen associations with cognitive impairment in the Panama Aging Research Initiative Study

**DOI:** 10.1002/alz70855_099154

**Published:** 2025-12-28

**Authors:** Giselle Rangel, Berta Alicia Muñoz, Morgan Ramirez, Kristen Wroblewski, Alcibiades E Villarreal, Diana Carolina Oviedo, Maria B Carreira, Rima McLeod, Gabrielle B Britton, Panama Aging Research Initiative Health Disparities (PARI‐HD)

**Affiliations:** ^1^ Instituto de Investigaciones Científicas y Servicios de Alta Tecnología (INDICASAT AIP), Centro de Neurociencias, Panamá, Panamá, Panama; ^2^ Sistema Nacional de Investigación (SNI), Panamá, Panamá, Panama; ^3^ Universidad de Panamá, Panama City, Panamá, Panama; ^4^ University of Chicago, Center for Global Health/ Toxoplasmosis Center and Toxoplasmosis Research Institute, Chicago, IL, USA; ^5^ University of Chicago, Chicago, IL, USA; ^6^ Escuela de Psicología, Universidad Santa María la Antigua (USMA), Panamá, Panamá, Panama

## Abstract

**Background:**

The prevalence of Alzheimer's disease (AD) in developing countries is expected to rise as a result of increases in life expectancy and the ageing of the population (1). Accumulating evidence implicates pathogens as triggers of inflammation and development of cognitive impairment and neurodegenerative disorders, including AD (2‐4).

**Method:**

This retrospective case‐control study aims to evaluate the association between cognitive impairment and specific antibodies status to diverse pathogens in a sample of 165 participants aged ≥65 years from the Panama Aging Research Initiative Health Disparities (PARI‐HD) study (5). Diagnosis was estimated during consensus review meetings by clinical experts in neurology, neuropsychology, and psychiatry. IgG antibodies against *Toxoplasma gondii*, Herpesvirus simplex type 1 (HSV‐1), Cytomegalovirus (CMV), *Helicobacter pylori, Chlamydophila pneumoniae, Treponema pallidum* and *Trypanosoma cruzi* were tested. Participant demographics, biomarkers of inflammation and cognitive‐functional factors were analyzed for associations with single/multiple pathogen‐specific antibodies reactivity using multivariable regression analyses.

**Result:**

Only *C. pneumoniae* seropositivity was significantly different between cognitively unimpaired and impaired groups (*p* = 0.02) and increasing TNF‐α levels were directly associated with C. *pneumoniae* seropositivity (OR=2.08, IC_95%_ 1.0‐4.1, *p* = 0.04). In addition, cumulative exposure to infectious agents increased the likelihood of cognitive impairment (OR=1.51, CI_95%_ 1.01‐2.26, *p* = 0.04) and poorer processing speed (OR=17.43, CI_95%_ 2.32‐32.53, *p* =  0.02). The presence of *C. pneumoniae* in multiple pathogen interactions significantly increased the likelihood of cognitive impairment diagnosis (OR=4.07, CI_95%_ 1.24‐13.36, *p* = 0.03).

**Conclusion:**

These results expand our understanding of cognitive impairment in a Hispanic population and warrant further studies on the role of C. pneumonia and multi‐pathogen infection in Alzheimer's disease.

